# “A steep learning curve”: junior doctor perspectives on the transition from medical student to the health-care workplace

**DOI:** 10.1186/s12909-017-0931-2

**Published:** 2017-05-26

**Authors:** Nancy Sturman, Zachary Tan, Jane Turner

**Affiliations:** 1 Primary Care Clinical Unit, Faculty of Medicine, University of Queensland, Brisbane, Australia; 2 Princess Alexandra Hospital and Faculty of Medicine, University of Queensland, Brisbane, Queensland Australia; 3 Discipline of Psychiatry, Faculty of Medicine, University of Queensland, Brisbane, Australia

## Abstract

**Background:**

The transition from medical student to hospital-based first year junior doctor (termed “intern” in Australia) is known to be challenging, and recent changes in clinical learning environments may reduce graduate preparedness for the intern workplace. Although manageable challenges and transitions are a stimulus to learning, levels of burnout in junior medical colleagues are concerning. In order to prepare and support medical graduates, educators need to understand contemporary junior doctor perspectives on this transition.

**Methods:**

Final-year University of Queensland medical students recruited junior doctors working in diverse hospital settings, and videorecorded individual semi-structured interviews about their transition from medical student to working as a junior doctor. Two clinical academics (NS and JT) and an intern (ZT) independently conducted a descriptive analysis of interview transcripts, and identified preliminary emerging concepts and themes, before reaching agreement by consensus on the major overarching themes.

**Results:**

Three key themes emerged from the analysis of 15 interviews: internship as a “steep learning curve”; relationships and team; and seeking help. Participants described the intern transition as physically, mentally and emotionally exhausting. They learned to manage long days, administrative and clinical tasks, frequent interruptions and time pressures; identify priorities; deal with criticism without compromising key relationships; communicate succinctly; understand team roles (including their own status within hospital hierarchies); and negotiate conflict. Participants reported a drop in self-confidence, and difficulty maintaining self-care and social relationships. Although participants emphasised the importance of escalating concerns and seeking help to manage patients, they appeared more reluctant to seek help for personal issues and reported a number of barriers to doing so.

**Conclusion:**

Findings may assist educators in refining their intern preparation and intern training curricula, and ensuring that medical school and intern preparation priorities are not seen as competing. Insights from non-medical disciplines into the organisational and relational challenges facing junior doctors and their health-care teams may enhance inter-professional learning opportunities. Workplace support and teaching, especially from junior colleagues, is highly valued during the demanding intern transition.

## Background

The transition from medical student to first year junior doctor (known as the ‘medical intern’ in Australia) is known to be stressful; it has indeed been described as a test of “fortitude and resolve” [1] and “a wilderness experience” [2]. Although surviving the day to day realities of being “thrown in at the deep end” [3] is believed to socialise the junior doctor and increase his or her confidence [2], reports of high levels of junior doctor anxiety [4] and burnout [5] are concerning. Many medical schools have responded to such reports by introducing intern preparation programs designed to reduce the abruptness of the transition [6], and there have also been a number of calls for more systematic guidance and training for both trainees and supervisors in the junior doctor years [1].

Transitions are embedded in medical training and practice, which has been described as having a somewhat “fragmented overall structure” [6]. The intern transition is the first of many workplace transitions for the junior doctor, who must continue to learn on the job while providing patient care. There are a number of theories of work-based learning [7, 8], and transitioning to new work roles [6]. Probably the best known theory of learning in healthcare settings cites these workplaces as examples of local “communities of practice”, based on their sustained relationships, common understandings and social norms, sense of belonging, and particular practical repertoires in which new recruits gradually gain proficiency through deliberate and supported practice [2, 8]. However it has been argued recently that current hospital healthcare settings may provide fewer opportunities for clinical year medical students and junior doctors to undertake this deliberate and supported practice, due to the relative isolation of learners from senior Faculty, rapid throughput of patients with diagnoses established prior to admission and fewer clinical signs than previously, inconsistent clinical exposure and increasing specialization [9, 10]. Frequent student and junior doctor transitions from one clinical team to another may also have a detrimental impact on professional identity formation (coming to “think, act and feel like a physician” [11]). Short stints in different clinical placements have been reported to promote shallow relationships with both patients and clinical teams, as well as a tendency to “manage (supervisors’) impressions” rather than seeking teaching from them, and find workarounds (including cutting corners and not answering pages) rather than investing in understanding and improving healthcare systems [9, 12]. It is therefore possible that medical students are currently graduating less well prepared for their junior doctor roles in terms of both knowledge and skills, and their developing professional identities. This may increase the abruptness and stressfulness of the intern transition.

Transitions are times of both risk and opportunity. Given that safe and effective patient care, and junior doctor well-being, are key priorities of the hospital workplace, preparation and support for the intern transition remain a key focus of medical education and training. Senior clinicians, whose evaluations of intern performance contribute to important decisions about speciality training programme selection and progression, may be quite removed from the workplace realities of their junior doctor colleagues. Educators and supervisors should understand the realities of contemporary intern life for medical graduates, and partner with them to optimise both undergraduate and postgraduate support and learning opportunities. The people who are likely to best understand the intern transition are recently graduated young doctors, who are also known to be key supports to their incoming colleagues [1].

The aims of this project were to better understand the intern transition, and evaluate how effectively our medical programme is preparing students for their intern roles, by seeking the perspectives of recent graduates.

## Methods

In this project, six final year University of Queensland (UQ) medical students (three male and three female) were recruited on a medical student Facebook social media site, and invited to conduct individual semi-structured interviews with junior doctors about their experiences of the transition from medical student to intern. Junior doctor participants were selected by student interviewers through their personal networks, with the aim of obtaining a diverse sample of junior doctors and intern workplaces. The students developed a semi-structured interview guide in a session facilitated by ZT, following a general description of the “Intern Voice” project goals. The interview guide developed by the students invited the junior doctors to reflect on four areas: 1) their views about the medical programme, including any areas for improvement; 2) their medical student experience, including any advice for current students; 3) life as an intern and how prepared they felt for this; and 4) how to stay resilient through medical studies and training.

Junior doctor interviewees were advised that excerpts of the interview might be selected by the investigators and presented to the student body and to academic staff involved in the medical program curriculum, but that they would be given a preview of the draft presentations with the right to exclude any excerpts in which they featured. All interviews were video-recorded, and the audio content was transcribed.

One of the investigators (ZT) was a final year medical student at the time of the interviews, and a medical intern at the time of the data analysis. The other two investigators are academic staff in the UQ School of Medicine involved in curriculum development and delivery, and clinical teaching (NS in a general practice setting, JT in a hospital-based mental health facility). All interview transcripts were read in their entirety by all three investigators independently to gain familiarity with the data. Salient phrases or comments which exemplified provisional concepts or themes were highlighted in each interview, and compared across interviews to refine or expand the preliminary themes which emerged [13]. The investigators met three times face-to-face to refine the analysis. Emerging concepts and themes were identified by consensus using an iterative, inductive approach with constant comparison between interview data and the investigators’ preliminary concepts [14]. Refined concepts were categorised into major overarching themes, for which illustrative quotations were identified [13].

The investigators developed two summaries of the key recommendations made by participants in relation to 1) improving the medical programme, which was presented at a Faculty Teaching and Learning Committee, and 2) making the most out of medical school, which was uploaded to student eLearning sites. This paper focuses on findings from interview questions about 3) life as an intern, and 4) resilience during studies and training.

## Results

The student interviewers recruited and interviewed fifteen junior doctors. A total of 12 h of interview footage was video-recorded. Participant demographics are shown in Table 1.

**Table 1:** Junior doctor participant demographics

	Gender	Age in years	Domestic or International student^b^	Previous highest undergraduate degree^c^	Junior doctor workplace^d^ Queensland (QLD) or International (Int)
Number of participants (*N* = 15)	Male: 11 Female: 4^a^	<21: nil 21–25: 3 26–30: 9 31–35: 3 >35: nil	Australian: 8 International: 7	BA: 2 BSc: 10 MSc: 1 Pharmacy: 2 Engineering: 1	QLD: 12 Int: 3

^a^The UQ medical student cohort is approximately 60% male, 40% female

^b^All international students were from Canada or USA. The UQ medical student cohort includes approximately 30% international students

^c^The UQ medical program accepts only graduate students. One student had completed both a BA and a BSc

^d^All international workplaces were in North America. Three Australian workplaces included regional and rural hospital placements

Interviewees appeared in the video footage to be engaged and relaxed in the interviews, although a minority requested ‘re-starts’ during their interviews to allow them to re-express their ideas more fluently. Although analysis commenced once interviews were completed, and saturation was not actively sought, no new themes emerged after the first ten interviews had been analysed.

Several participants made a number of specific suggestions in order to improve intern preparation, although others were sceptical that more effective preparation was possible. More teaching in a number of procedural, technical, clinical, communication and relational areas (including lumbar puncture, completing forms, managing ward call scenarios, writing in medical records, and negotiating conflict) was recommended. Medical students were advised to engage actively on the wards, and shadow their interns during their daily activities and during ward call shifts, in preference to spending time shadowing senior consultants or studying for medical assessments. Participants reported having experienced competing priorities in terms of performing well in medical school assessments, and engaging with their clinical teams; they reported having appreciated clinical rotations with lighter assessment loads.

Three key overarching themes emerged from the analysis: internship as a steep learning curve; the importance of relationships and working in teams; and responding to challenges and seeking help. The preliminary concepts of stepping up, changing focus, endurance, exhaustion, responsibility for patients, impact on senior colleagues, mistakes, criticism, self-care and self-doubt fell under “a steep learning curve”; communication, inter-professional roles, hierarchy, trust, professionalism, conflict, and being strategic with professional relationships fell under the relationships and team over-arching theme; and escalating, prioritising, being organised, peer support, family and friends, life outside medicine, stigma, and help-seeking fell under the overarching theme of responding to challenges and seeking help.

Illustrative quotations are provided in Table 2, for each of these themes.

**Table 2:** Illustrative quotations for major themes

Theme One: A steep learning curve
I3P1 *It’s an incredibly steep learning curve, it’s – you can say the same at any workplace – it’s any new job, any new role, it’s going to be vastly different to what you’ve done as a student, but I think it’s – it was a huge learning curve and it was a huge shock to the system when – everyone that I’ve talked to, it was a huge learning curve for everyone.*
I1P2 *It’s long hours and you’re tired and trying to find time to sleep and exercise...also just have (*sic*) a clear mind on a really foggy head is a challenge and just getting used to the demands physically, emotionally for sure, mentally with this job.*
I4P1 *There’s incredible self-doubt when you become an intern that you don’t have as a medical student. So I think going from being the top of your game as a medical student to the absolute bottom of the ladder as an intern is a huge transition emotionally in terms of ego and it’s a big knock to your confidence.*
I5P2 *Some of the biggest challenges would be I guess taking care of sick patients. So you’re a junior doctor, it’s your first year practising, you can get very nervous when a patient’s blood pressure drops, they look very sick and unwell.*
Theme Two: Relationships and team
I5P3 *A lot of the times you will have registrars and consultants who are quite over bearing and might be quite aggressive and harsh in terms of their criticisms and how they want you to do things. I think the only way really to deal with that is just to take it. Because I mean these guys are your bosses, they’re going to be the ones deciding whether you get into a certain college or anything like that and you really need to build a resilience to that and just take any criticism. There’s no need to talk back or argue back.*
I6P2 *Sometimes other professionals, like nursing on occasion or allied health, will maybe disagree or raise concerns with a plan that we have and what I’ve found is that it’s very easy for their concerns to be raised with us, but when we as junior – because we are junior doctors – have concerns about perhaps the way other professions are managing our patients, I find it challenging to find the right avenues...I’ve had experiences where my concerns have been dismissed and I feel like I’ve not been listened to in terms of the thing that I was worried about....you’re both at the bottom and somewhere up in the middle of the food chain at the same time because you are making decisions and directing patient care, but at the same time you’re at the very, very bottom of that chain, so that’s a bit of a confusing situation to be in especially when you have disagreements with other staff.*
I5P1 *As a resident you are relaying the information about your patients to numerous teams and numerous different professionals every single day and if that’s a skill that’s not up to scratch, you’ll have a lot of difficulty organising anything for your patients, or getting anything done it will be very difficult...you need to be prepared and you need to have a way of sending your message succinctly and to the point.*
I3P1 *Working in a team, it’s something that you have to learn to do, it’s something that you don’t do well to begin with and something you have to learn to do because no matter what you do in a hospital setting you’re going to be part of a bigger team...and you have to learn to work with a lot of different personalities and a lot of different work ethics and things like that, which is not something that can be taught as easily and it’s something that comes more with experience and time in just being in that setting.*
Theme Three: Responding to challenges and seeking help
I5P1 *The most challenging times will come when you’re on ward call or you’re on - doing anything that’s by yourself...and the most important thing that you should be thinking about is, when do I need to escalate this problem that I have with a patient to someone more senior.*
I3P1 *The advice that I would give is don’t be afraid, everyone is there to help you and everyone is there to guide you along, don’t be afraid to ask questions because ultimately it’s all in the best interest of the patient.*
I2P1 *Internship is extraordinarily stressful and I think it brings out a lot of issues that people have, and I think some of those issues could be dealt with much earlier on....If they’re having a difficult time even in school, it’s likely that they’re going to have a difficult time in internship as well.*
I1P3 *The state medical boards will want to know if you ever sought mental health treatment, so that’s a huge barrier. My insurance, if I were to seek medical mental health assistance, a lot of that would be through here which are people I work with which isn’t necessarily ideal. And there is a stigma, it’s like you’re not hard enough, you’re not able to carry your weight and having to take – and there’s also very real fact of the matter is if I’m sick or I need to take time off or something there’s other people that’s going to have to carry my weight while I’m gone, so you do have that pressure to keep pushing forward, keep pushing forward no matter what.*

### Theme one: a steep learning curve

Participants described the transition to internship as physically, psychologically, cognitively and emotionally exhausting. They also described an abrupt change in focus from what had mattered as a medical student to their new priorities as an intern, and an abrupt change from self-confidence to self-doubt and uncertainty. They reported learning to manage time pressures, frequent interruptions, changing priorities, long days, mistakes and criticism. Each clinical context was described as having its own local systems, which had to be learned by experience. The responsibility of caring for sick patients appeared to be felt keenly. Several participants reported difficulty maintaining self-care activities and social relationships especially outside work.

### Theme two: relationships and team

Participants reported that a supportive team, and teaching and support from junior colleagues, were key in negotiating the intern transition. They reported some challenges working in teams, including identifying roles and negotiating different work ethics, personalities and approaches to patient care. Participants reported receiving criticism, some of it felt to be harsh, from other team members, both medical and non-medical. They were aware of the importance for their future medical careers of maintaining positive relationships especially with more senior medical colleagues. Participants described having a somewhat paradoxical status in hospital hierarchies, and emphasised the importance of communicating succinctly and accurately.

### Theme three: responding to challenges and seeking help

Participants reported responding to the challenges by learning to be organised in managing administrative and clinical tasks, identifying and acting on priorities, and having a low threshold for escalating concerns. Participants emphasised the importance of actively consulting resources and seeking help to manage patients safely, but appeared to be less comfortable seeking help for their own personal or health problems. Participants suggested that pre-existing problems (for example depression which had developed during the medical program) were likely to be exacerbated during internship. They urged any students or junior doctors who were not coping to access help, but none admitted having sought help other than from family and friends themselves, and there was a strong view that obtaining professional assistance was complex because of stigma, time pressures, reluctance to increase the workloads of colleagues, and on occasion feelings of helplessness. Participants admitted to limited knowledge of specific services or resources which might be available.

## Discussion

The intern transition is a key opportunity for senior medical students and junior doctors to “learn to play the role of physician, acquire the language of medicine, understand the hierarchy of the profession and its power structures, and learn how to live with ambiguity” [15]. Our findings suggest that the intern transition is experienced as abrupt and stressful, but affords many learning opportunities. Our findings extend previous reports by identifying many of the specific challenges involved, and reporting some junior doctor recommendations for improving intern preparation.

Although the team-based nature of health-care is emphasised in many current accounts of effective medical practice, our data provide some insights into the contemporary junior doctor perspective of working in health care teams.

The use of near peers as interviewers, who were personally known to participants, was a deliberate strategy to obtain candid reflections in a context of trust. This strategy also aligns well with educational models which view students and recent alumni as partners with academic teams in curricular design and evaluation [16]. However participants were aware that their comments might be selected for wider dissemination and might have had some reservations about divulging attitudes which had the potential to be perceived by academic staff as negative. The assurance that any video footage of such comments would be previewed by them, and would be excluded at their request, was intended to reassure participants who might have such concerns. Although participants were graduates of a single institution, purposive sampling ensured that data captures a diverse range of intern experiences. Three participants were working in North American settings, which differ in several respects from the Australian setting, including the streaming of interns into specialty training immediately after graduation, and different systems of health insurance. These participants are not identified because of the small numbers and concern about preserving anonymity.

Our participants reported being relatively well prepared in terms of clinical skills and knowledge (or at least able to access additional knowledge as needed). However, as other investigators have also found [3], they reported feeling under-prepared for management and administrative skills, and medical educators should consider explicitly discussing the more technical aspects of the intern role to prepare students for the fact that administrative tasks form a large part of their working day. Although active participation in their hospital communities of practice, and especially junior doctor shadowing, have previously been identified as particularly valuable for intern preparation [1, 2], participants reported having felt pressured to spend time with more senior clinicians instead. They were very aware of the potential negative impact on their careers of poor relationships with senior doctors.

Team support has also been recognised as critical in previous literature, particularly teams which show interest in the junior doctor as an individual [3] and provide regular feedback [1]. Shadowing junior doctors and sharing experiences with peers have also previously been identified as particularly valuable [12, 17]; this sharing may promote the “strong sense of shared social identity” which is known to be a factor in buffering stress [11]. Our participants recognised the importance of working well with other team members, but commented frequently on difference and conflict within these teams. They also acknowledged the paradoxical position of the junior doctor within the hospital hierarchy (which has received little discussion in previous literature), and acknowledged that it could be difficult to identify the roles played by other team members. This latter difficulty has previously been identified, and junior doctors have been described as “working in parallel with other team members” rather than together [12], with no shared vision of care [2]. Inter-professional education in simulated settings aims to challenge pre-existing attitudes towards different health professional groups (for example perceptions that some are more caring than others [18]), and prepare students to function in healthcare teams. However it has been suggested that team membership may be meaningless to students, until they start to feel useful [2]. Our findings suggest that inter-professional education should also support students to learn to negotiate differences in goals and attitudes towards work, difficult personalities, criticism and conflict within teams. More sophisticated preparation for organizational, team and relational challenges may benefit from insights from disciplines outside the health sciences including the social and behavioural sciences.

Participants described an initial loss of self-confidence during the intern transition, when faced with the responsibility and the uncertainty of real-world clinical practice. However they reported having adjusted quickly to the new demands of the junior doctor workplace, and appeared reluctant to report ongoing challenges. This is in keeping with previous findings that interns take some pride in surviving without “needing their hands held” [12]. Previous investigators have suggested that “feelings of vulnerability are often hidden and frequently not articulated” [19], and that more advanced junior doctors downplay the challenges of the intern transition [12].

Our participants emphasised the importance of finding individual strategies to cope with workplace stress, including exercise, socializing with both medical and non-medical friends and family, and various leisure activities. Participants emphasised the importance of seeking clarification, advice and support in managing patients. Seeking help, and escalating appropriately, are of course essential for patient care: skills in “slowing down”, and “help seeking” when appropriate, have a major impact on patient safety [20]. Medical educators have also emphasised the importance of seeking external feedback (“habits of self-directed assessment seeking”) to enhance learning and self-monitoring [20, 21]. However our participants did not report seeking help to cope with stress or personal health problems, despite recommending that others do so. Doctors’ decisions to access healthcare for themselves are complex: they are taken in the context of being time poor, learning to dismiss minor symptoms during medical school, clinical experience managing symptoms expectantly and tolerating uncertainty, setting a high value on self-management of health conditions, and an awareness of the psychological basis of many physical symptoms [22]. Stigma was also highlighted as a significant barrier, as was the potential for a longer term negative impact on career progression. Given these challenges it may be particularly important to facilitate access to healthcare for both medical students and junior doctors by signposting appropriate points of entry (including building relationships of trust with independent and caring general practitioners).

## Conclusions

The intern transition continues to be experienced as abrupt, stressful and exhausting; our participants reported a drop in self-confidence, and difficulty maintaining self-care and social relationships. We extend previous reports by identifying many of the specific challenges involved, from the perspective of the junior doctor. Participants reported learning to manage long days, administrative and clinical tasks, frequent interruptions and time pressures; identify priorities; deal with criticism without compromising key relationships; communicate succinctly; understand team roles (including their own status within hospital hierarchies); and negotiate conflict. Supportive clinical teams, and teaching and support from junior colleagues, are reported to be invaluable in negotiating these challenges.

Findings may assist educators in refining their Intern preparation and intern training curricula, and ensuring that medical school and intern preparation priorities are not seen as competing. Inter-professional educators may find it useful to reflect on some of the team-based challenges which junior doctor participants identify, and consider including teaching from disciplines outside medicine and the health sciences about the organisational and relational challenges facing junior doctors and their health-care teams. Although junior doctor participants appeared to be comfortable seeking help to manage patients, they may be more reluctant to seek help to manage personal problems and report a number of barriers to doing so. Junior doctors are also very aware of the importance of maintaining positive relationships with senior medical colleagues.
